# The Role of GPNMB in Inflammation

**DOI:** 10.3389/fimmu.2021.674739

**Published:** 2021-05-12

**Authors:** Marina Saade, Giovanna Araujo de Souza, Cristoforo Scavone, Paula Fernanda Kinoshita

**Affiliations:** Department of Pharmacology, Institute of Biomedical Science, University of São Paulo, São Paulo, Brazil

**Keywords:** GPNMB, inflammation, neuroinflammation, macrophages, cancer, protection

## Abstract

Inflammation is a response to a lesion in the tissue or infection. This process occurs in a specific manner in the central nervous system and is called neuroinflammation, which is involved in neurodegenerative diseases. GPNMB, an endogenous glycoprotein, has been recently related to inflammation and neuroinflammation. GPNMB is highly expressed in macrophages and microglia, which are cells involved with innate immune response in the periphery and the brain, respectively. Some studies have shown increased levels of GPNMB in pro-inflammatory conditions, such as LPS treatment, and in pathological conditions, such as neurodegenerative diseases and cancer. However, the role of GPNMB in inflammation is still not clear. Even though most studies suggest that GPNMB might have an anti-inflammatory role by promoting inflammation resolution, there is evidence that GPNMB could be pro-inflammatory. In this review, we gather and discuss the published evidence regarding this interaction.

## Inflammation

Inflammation is a complex response to microbial infections or tissue damage involving interactions between soluble molecules and cellular effectors to restore homeostasis (1–3). Tissue-resident macrophages and mast cells are relevant to an efficient and rapid immune response that prevents the infection from spreading and/or tissue damage. Those cells can monitor other cells and tissues by sensing any disbalance in homeostasis (3).

Macrophages are phagocytic cells of the innate immune system which are responsible for recognizing an infection, presenting antigens, and also for the production of inflammatory mediators, the removal of apoptotic cells, and the output of growth-factors (1, 4). Macrophages can be polarized in two different subsets that differ in cytokine production, iron, and glucose metabolism (5). The subtype called M1 is activated by pro-inflammatory cytokines or microorganism’s molecules (such as LPS, a lipopolysaccharide and component of gram-negative bacteria membranes), and has killing capacity by the production of reactive oxygen species (ROS), nitric oxide (NO), and inflammatory cytokines (6). M2 macrophages are activated by anti-inflammatory cytokines like interleukins (IL-4, IL-13, IL-10), transforming growth factor-beta (TGF-β), and glucocorticoids. These types of cells establish a healing or growing setting promoting tissue remodeling, angiogenesis, and parasite clearance (7). However, these two macrophages profiles are a simplified version, found in *in vitro* studies. *In vivo*, there is a range of functional subsets within M1-M2 extreme profiles, which can be adapted depending on the microenvironment the cells are inserted in (7).

The activation of the immune system starts with the recognition of some patterns known as pathogens-associated molecular patterns (PAMPs) or damage-associated molecular patterns (DAMPs) by the effector cells of the innate immune system like macrophages, monocytes, and dendritic cells (1, 2). PAMPS are molecules essential for microbial survival. Some examples of PAMPs are nucleic acid, e.g., single-stranded RNA of viruses, plasmatic membrane compounds, or cell walls, e.g., LPS in gram-negative bacteria and b-glucans in fungus (4). DAMPs are endogenous and released due to damage, such as heat-shock proteins, uric acid crystals, serum amyloid A, and nucleic acid fragments (8).

PAMPs and DAMPs are recognized by pattern recognition receptors (PRRs), a set of signaling receptors on transmembrane or intracellular compartments (9). An essential class of PRRs is Toll-like receptors (TLRs), a family of nine transmembrane proteins that activate the expression of transcription factors such as nuclear factor kappa-light-chain-enhancer of activated B cells (NF-kB) and interferon regulatory factors (IRFs) (4).

NF-kB is a protein complex expressed constitutively in the cytoplasm that is sequestered by IκB. The translocation of NF-kB to the nucleus is dependent on IκB phosphorylation by IκB kinases (IKK). In pro-inflammatory conditions, the NF-kB dimer activated is composed of p65 (RelA) and p50 subunits, which modulate the transcription of pro-inflammatory genes and the adaptive immune system signaling (2). The production of pro-inflammatory mediators, such as cytokines (tumor necrosis factor (TNF), IL-1, IL-6, and interferon-gamma (IFNγ), chemokines, vasoactive amines (histamine and serotonin), and eicosanoids, recruit effector cells of the acute immune response like monocytes and neutrophils to the site of inflammation, which promotes some damage to the infector agent and may eliminate it (1).

In the early stages of the inflammatory reaction, there is an important loss of tissue-resident macrophages due to cell death. To reestablish this population of cells, two approaches arise recruitment of monocytes and enhancement of tissue macrophage proliferation by their self-renewal ability (7). Although monocytes and macrophages have similar functions during inflammation, they differ from each other in origin. Monocytes originate from the bone marrow, and macrophages from the yolk sac, fetal liver, and bone marrow. The differences also appear in some functions beyond immune response and phagocytosis, since monocytes also patrol and monitor the luminal surface of the endothelium, and macrophages play key roles in tissue development, surveillance, and monitoring of tissue changes, and maintenance of tissue homeostasis (7). They also differ in the localization aspect. Monocytes circulate in the blood, bone marrow, and spleen (7), and macrophages are resident-tissue cells (10, 11).

Different from the innate response, the adaptive response is much more specific and refined not only for the recognition of the antigen, but also for its effector response. The principal cells involved in this response are lymphocytes: T cells and B cells (9).

After the end of the infection, anti-inflammatory cytokines as IL-10 and TGF- β are secreted to promote the resolution of the immune response. The resolution and repair phases are mediated by macrophages (1). The resolution of inflammation is as important as its activation, allowing the cells to return to homeostasis.

## Neuroinflammation

The central nervous system (CNS) is an immune-privileged tissue, protected by the blood-brain barrier. Hence, peripheral immune cells do not have access to the brain in physiological circumstances. Besides microglial cells, other macrophages are found in external parts of the CNS, in meninges, choroid plexus, and perivascular spaces. Moreover, other immune cells like dendritic cells, monocytes, and astrocytes can be found in CNS (12). This privileged feature confers a differentiated inflammatory response, which is called neuroinflammation.

Neuroinflammation is present in many disturbances of the CNS, including neurodegenerative diseases, such as Alzheimer’s disease (AD), Amyotrophic lateral sclerosis (ALS), Multiple sclerosis (MS), Parkinson’s disease (PD), and Frontotemporal dementia. Although it is not known if neuroinflammation is the initial cause of neurodegenerative diseases, neuroinflammation promotes a prolonged activation of microglia and astrocytes and is an important factor in disease progression (13, 14). Neuroinflammation also exhibits important roles in the maintenance and progression of diseases, leading to neurotoxicity, neural death, and associated symptoms.

Microglia is a tissue-resident macrophage in the CNS. Similar to peripheral macrophages, microglia plays an important role in environmental surveillance, homeostasis maintenance, and response to any type of disbalance (12, 15). Depending on the type of stimulus secreted by the surrounding cells, different microglia phenotypes are activated, leading to distinct responses. Originally, the microglial activation was classified, the same way as macrophages, in two states: M1 (classically activated) and M2 (alternatively activated). M1 is considered a deleterious and pro-inflammatory state for ROS production and release of inflammatory molecules such as TNF-α, IL-1β, IL-12, whereas M2 is classified as an anti-inflammatory state, that is involved in the resolution of inflammation, the release of trophic factors like TGF-β and brain-derived neurotrophic factor (BDNF) (16, 17). This classification is considered outdated once a variety of microglia phenotypes were found in the brain, and whose activation states are beyond these extreme M1-M2 classifications.

After activation, microglia can play different roles including antigen presentation, phagocytosis, secretion of pro- and anti-inflammatory mediators, and production of neurotrophic factors (18). After activation *via* PRR, different signaling pathways like NF-kB, JAK/STAT, ERK1/2, AP-1, and p68 are responsible for targeting genes to promote the production of cytokines (TNF, IL-6, IL-1β, and IL-18), chemokines (CCL2, CCL3, CCL4, and CCL7), and other molecules like ROS, reactive nitrogen species and glutamate (19). Microglia also produces anti-inflammatory mediators like IL-4, IL-10, IL-13, and TGF-β (12). In this scenario, microglia appears to be the common initial sensor of damage, thus establishing the inflammatory state, and afterward amplification by astrocytes.

In neurodegenerative diseases, a specific type of microglia was found, subsequently classified as disease-associated microglia (DAM) characterized by transcriptional changes driven by apolipoprotein E (APOE) and the triggering receptor expressed on myeloid cells 2 (TREM2) (20). Studies demonstrate that the DAM has a molecular program with upregulation of genes that encode inflammatory molecules such as *Itgax*, *Ccl2*, and downregulation of homeostatic genes like *Csf1r*, *Cx3cr1*, *Hexb*, and *Trem119* (20). Glycoprotein nonmetastatic melanoma protein B (GPNMB) is also a molecule that has been described to be upregulated in DAM (20, 21).

## GPNMB

GPNMB is an endogenous type 1 transmembrane glycoprotein that was first described in 1995 as more highly expressed in low-metastatic melanoma cell lines (22). GPNMB is also known as Osteactivin, DC-HIL (dendritic cell heparan sulfate proteoglycan integrin-dependent ligand), and HGFIN (hematopoietic growth factor inducible neurokinin-1 type). While the name GPNMB is more broadly used, the name Osteoactivin is mostly used in studies with bones, while DC-HIL and HGFIN are commonly used in studies about the immune system and cells. GPNMB is constitutively expressed through most cell types and tissues (23), and its expression can be increased in cancer cells (24).

GPNMB is encoded by the GPNMB gene located at locus 7p15 and has 2 isoforms: one with 572 and the other with 560 amino acids, as a result of alternative splicing (25). After transcription, GPNMB is directed to the cell membrane, in which most of its length is located in the extracellular domain. The extracellular portion of GPNMB has 12 glycosylation sites, a polycystic kidney disease (PKD) domain, and an integrin-recognition (RGD) motif (26). GPNMB can be cleaved by the metalloproteinase ADAM10, releasing a soluble fragment that can bind to many receptors and trigger a cellular response (27).

GPNMB is known to have different functions on different cell types. In bones, GPNMB promotes differentiation of osteoblasts and mineralization of bone matrix (26), fibroblast activation and proliferation (28), maturation of hematopoietic and lymphoid cells (29), and also decreases the activation of lymphocytes T (30). The broad range of GPNMB’s extracellular fragment function is related to its ability to bind to many receptors, such as Na^+^, K^+^-ATPase (NKA), CD44, Epidermal Growth Factor Receptor (EGFR), and Vascular Endothelial Growth Factor Receptor (VEGFR) (24) and its ability to interact with integrins, heparin and Syndecan-4 (31, 32).

**Figure 1:**
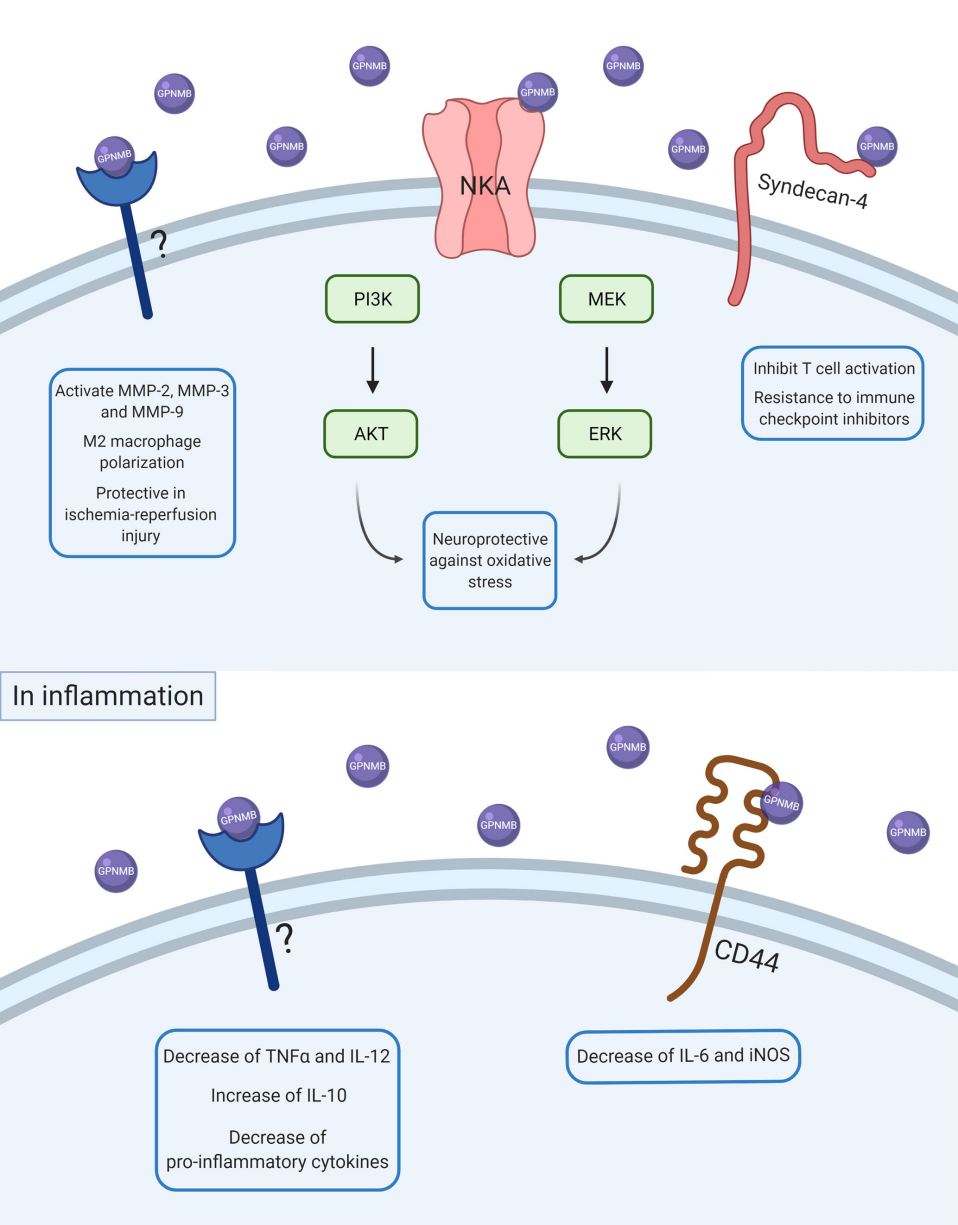
Scheme of GPNMB intracellular signaling in physiological and inflammatory conditions. After ADAM10 cleavage, soluble GPNMB can interact with several receptors, proteins, and other molecules in the cell membrane. Some of these interactions can activate intracellular pathways and lead to changes in other proteins’ expression and consequently in cell response. In basal conditions, GPNMB can interact with NKA and promote neuroprotection against oxidative stress by activation of ERK/MEK and AKT/PI3K pathways. GPNMB can also interact with syndecan-4 and promote inhibition of T cell activation, which is also associated with a resistance of immune checkpoint inhibitors. GPNMB can also promote activation of MMP-2, MMP-3, and MMP-9, M2-macrophage polarization, and a protective role in ischemia-reperfusion injury, but the molecular mechanisms underlying these responses are not known yet. In inflammation, GPNMB seems to have an anti-inflammatory and resolute role by decreasing pro-inflammatory cytokines like TNFα, IL-6, and IL-12 and increasing anti-inflammatory cytokines such as IL-10.

As illustrated in **Figure 1**, the interaction between GPNMB and the NKA activates the ERK/MERK and Akt/PI3K pathways (33, 34), which can modulate the activation of NF-κB (35, 36). Previous studies have shown an increase in phosphorylation of ERK and Akt by GPNMB signaling in different models and cell types (33, 34, 37, 38). The Akt residue that appears to be phosphorylated by GPNMB is S473 (34, 38), required for the maximum activation of the kinase (39).

As GPNMB was originally associated with tumorous cells, GPNMB was widely studied in several types of cancer, such as melanoma (40), glioma (41), breast cancer (42), and gastric cancer (43). Although previous work indicated that GPNMB is an anti-metastatic glycoprotein, recent studies question the function of GPNMB in cancer, because GPNMB appears to have a protective role in some types of cancers whereas it may promote metastasis in others (24). In the past years, GPNMB has been studied in breast cancer, whose role has been controversial for years, but most of the evidence suggests that GPNMB is associated with cancer aggressiveness, as it promotes cancer metastasis (42), angiogenesis (27) and immune suppression (40, 44, 45), enhancing tumor migration and invasion. An antibody anti-GPNMB (called CDX-011 or glembatumumab vedotin) has been evaluated in phase I/II trials as a treatment for advanced breast cancer and melanoma (46–48). Clinical trials were discontinued in 2018 after glembatumumab vedotin failed to improve the survival rate and stop tumor progression of women with metastatic triple-negative breast cancers (TNBC) for longer than capecitabine (49).

More recently, the relationship between GPNMB and inflammation has been explored in a few studies, but the function of GPNMB in inflammation is still not clear, as some studies suggest anti-inflammatory action of GPNMB, while others suggest that it acts as a pro-inflammatory mediator (**Table 1**). In this review, we will gather recent studies about this subject to better understand the relationship between GPNMB and inflammation in different systems.

**Table 1:** Factors that modulate GPNMB expression.

	Condition/Treatment	Model	Reference
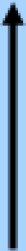	LPS	i.p. injection (increase in the brain)	(50)
		*in vitro* treatment (BV-2 cells)	(51)
		*in vitro* treatment + IFN-γ (BMM cells)	(52)
	Alzheimer's disease	amyloid beta treatment; patients	(21)
	Parkinson's disease	SNP rs199347; patients	(53, 54)
	Amyotrophic Lateral Sclerosis	SOD1 murine models; patients	(33, 34)
	Cancer	patients, *in vitro* models; murine models	(40–44)
	Ischemia-reperfusion injury	murine model of cerebral IRI	(37)
		murine model of liver and kidney IRI	(55)
		murine model of kidney IRI	(56)
	Liver damage	CCl4 administration *in vivo*	(57)
		patients (hepatitis, cirrhosis, etc.)	(57)
	End-stage renal disease	patients	(58)
	Niemann-Pick type C disease	patients and murine model	(59)
	Gaucher disease	patients	(60, 61)
		murine model	(60)
	Colitis	mice model	(62)
	M2-polarized macrophages	*in vitro*	(38, 56)
	IL-4	*in vitro* treatment	(38, 54)
	Obesity	murine model	(63)
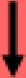	LPS	*in vitro* treatment (mononuclear phagocytes)	(57)
	Amyloidosis Cutis Dyschromica	mutation on the GPNMB gene	(64)

The up arrow group (↑), in blue, gathers evidence that suggests factors that lead to the upregulation of the GPNMB gene or/and increased levels of soluble GPNMB. The down arrow group (↓), in red, are studies that suggest factors that decrease GPNMB expression.

## GPNMB and Neuroinflammation

GPNMB is expressed in all brain regions, but especially in the hippocampal dentate gyrus, choroid plexus, ependyma, periventricular regions, and in layers II and III of the cerebral cortex (50). Besides the brain region, GPNMB is mostly expressed by microglial cells, which are resident brain cells most responsible for mediating inflammatory stimuli and maintaining cerebral homeostasis by phagocytosis, tissue repair, and cytokine release (68).

Bandari and colleagues first described that GPNMB might be related to inflammation. It was shown that GPNMB could interact with Substance P (29), a peptide that facilitates inflammatory response in both the CNS and the Peripheral Nervous System (PNS) by interacting with the receptor NK1. The interaction between substance P and NK1 receptor promotes the activation of mTOR, which regulates inflammation by controlling the balance between IL-10 and IL-12, and the transcription factors AP-1 and NF-κB, which can increase the expression of many chemokines, leading to the promotion of recruitment of immune cells to sites of inflammation (69).

Additionally, there is evidence that pro-inflammatory stimuli could modulate GPNMB expression in the brain. An established model used to induce inflammation and neuroinflammation is the administration of LPS. Studies revealed an increase of pro-inflammatory cytokines released such as IL-1β, TNF-α, and IL-6, and also the activation of microglia in *in vitro* and *in vivo* studies after administration of LPS (70, 71). LPS could also disrupt BBB (72). It is important to underline that the route of administration, its dose, and timing are crucial for producing inflammatory changes (73).

Intraperitoneal (i.p.) injection of LPS in rats increases the number of cells expressing GPNMB, especially in the area postrema, which correlates with OX42 expression (50). OX42 is a marker present in both microglia and macrophages. As the area postrema is a region close to ventricular organs, the study suggests that the increase of GPNMB positive cells is due to increased infiltration of macrophages expressing GPNMB in the brain. Even though i.p. injection of LPS resulted in increased GPNMB levels in the brain, the same is still controversial *in vitro*. Two studies have treated BV-2 cells (microglia cell line) with LPS, but the outcome on mRNA levels of GPNMB was different. One study evidenced increased mRNA levels of *Gpnmb* after 6h of 10ng/ml or 100ng/ml LPS treatment, and in the 100ng/ml group, G*pnmb* mRNA levels remained increased for 12 and 24h. Besides, they have seen an increase in GPNMB protein expression after 12h in both LPS concentrations (51). In the other study, *Gpnmb* mRNA levels remained the same after 24h treatment with 100ng/ml of LPS, contradicting previous results (21). In one study, BV-2 cells were kept in Dulbecco’s modified Eagle’s medium (DMEM) supplemented with 10% fetal bovine serum (FBS), while in the other study, BV-2 were kept in DMEM/F12 media supplemented with 10% FBS.

An increase of GPNMB levels in the brain has also been detected in a diverse number of neurodegenerative diseases (*in vitro*, *in vivo*, and patients) such as AD (21), ALS (33, 34) and PD (53). Treatment with β-Amyloid (Aβ), a protein that accumulates in AD and forms aggregates, led to increased levels of *Gpnmb* mRNA expression. Interestingly, the increase of GPNMB occurs in microglia cells that are surrounding Aβ aggregate plates (21). In humans, it was shown that GPNMB was increased in cerebrospinal fluid (CSF) and post-mortem brains of sporadic AD patients. Sporadic ALS patients also present increased levels of GPNMB in CSF, serum, and lumbar spinal cord tissue (33). Increased levels of GPNMB expression were found in the substantia nigra of PD patients (54). Furthermore, the single nucleotide polymorphism (SNP) rs199347, a known top risk SNP for PD, is located in the GPNMB gene and results in increased GPNMB expression (74). This data supports that GPNMB might be considered a marker for DAM (20, 21).

In addition to being highly expressed, GPNMB seems to have a protective role in neurodegenerative diseases (**Table 1**). Although the mechanism underlying this protection is not yet well elucidated, it might be related to neuroinflammation. In a cellular ALS model, it has been shown that SOD1^G93A^ inhibits the glycosylation of GPNMB, resulting in increased motoneuron death (33) and treatment with recombinant extracellular fragments of GPNMB (rGPNMB) leads to diminished cell death (33, 34). The changes in cell viability can be due to GPNMB binding to NKA, which, besides maintaining the osmotic equilibrium of the cell, is a receptor known to modulate neuroinflammation (75, 76).

Also in the CNS, GPNMB seems to be protective in cerebral ischemia-reperfusion injury (IRI) (37), which is a condition known to trigger neuroinflammation (77). In this study, a transgenic mice overexpressing GPNMB was generated by the injection of a V5-His-tagged GPNMB cDNA construct containing a CAG hybrid promoter into fertilized mice eggs. Transgenic mice overexpressing GPNMB have resulted in decreased infarct volume after IRI and intraventricular administration of rGPNMB resulted in lessened cerebral infarct damage (37). Increased levels of GPNMB in neurons and astrocytes of both mice and humans were observed during the acute phase of IRI, suggesting that the response to inflammation could be modulated by GPNMB (37). In astrocyte primary culture, it has been evaluated that the administration of rGPNMB, in interaction with the CD44 receptor, leads to a decrease of IL-6 and iNOS levels that were induced by a pro-inflammatory cytokine-mix treatment (54).

Although most of the results suggest that GPNMB has an anti-inflammatory role, there is evidence that neuroinflammation could be dependent on GPNMB (**Table 1**). The silencing of GPNMB by transfection with GPNMB siRNA in BV-2 cells resulted in decreased levels of pro-inflammatory markers after LPS treatment (51). The increase of iNOS, TNF-α, and IL-1β levels was smaller in the group treated with LPS and GPNMB siRNA in comparison to cells treated with LPS with normal levels of GPNMB (51). Interestingly, phosphorylation of ERK1/2, which is increased with LPS treatment in normal cells, was also diminished in the GPNMB siRNA group treated with LPS. These results led to the conclusion that the induction of neuroinflammation *via* LPS could be dependent on GPNMB. Another evidence that contradicts previous results is that treatment with the anti-inflammatory cytokine IL-4, but not with a mix of pro-inflammatory cytokines, led to increased levels of GPNMB in astrocytes and mesenchymal cells (38, 54). These contradictory findings highlight that the relationship between GPNMB and neuroinflammation still needs to be more explored and is probably cell-dependent.

## GPNMB in Peripheral Inflammation

GPNMB is physiologically expressed in most cell types, and pro-inflammatory stimulus can cause its increase in some cell types, especially on macrophages and monocytes (**Table 1**). Microarray analysis evidenced GPNMB as a gene highly expressed in an inflammatory macrophage population (TEMP) compared with a non-stimulated macrophage population (BMM) or fibroblasts, and the administration of pro-inflammatory stimulus as LPS and IFN-γ led to increased levels of GPNMB in BMM (52).

In the liver of rats, GPNMB is constitutively expressed in peritoneal macrophages and Kupffer cells (resident liver macrophages) rather than in other cell types (57). Liver damage seems to increase GPNMB levels, as the induction of liver damage by CCl_4_ administration *in vivo* (57) resulted in higher levels of GPNMB. In human liver samples, GPNMB was not found in basal conditions but GPNMB is expressed in livers presenting hepatitis, cirrhosis, and paracetamol intoxication, which all refer to inflammatory diseases (57, 63). This data suggests that even if the GPNMB expression is different between rats and humans, the increase of GPNMB in a pro-inflammatory scenario is consistent.

GPNMB seems to be overexpressed in macrophages in diseases that have a pro-inflammatory component in different tissues. In colitis, a chronic inflammatory disease in the intestine, infiltrating macrophages in the injured mucosa express GPNMB, and GPNMB expression increases according to the severity of the illness (62). Interestingly, D2 mice, which is a mice strain known to have a premature stop codon mutation in the GPNMB gene, had a more severe condition of colitis, accompanied by increased levels of pro-inflammatory cytokines (62) (**Table 2**). In end-stage renal disease, a disease in which both inflammatory and mineralization processes are upregulated, patients have upregulation in G*pnmb* mRNA levels and protein expression in adhering monocytes, which was sustained and even increased after differentiation to macrophages *in vitro* (58). In addition, GPNMB is highly expressed in a murine model of IRI in the liver and kidney (55). GPNMB is also induced and secreted in white adipose tissue in obesity (63). Besides being increased in disease, it has been reported in humans that mutations that result in truncation of the GPNMB gene are linked to Autosomal-Recessive Amyloidosis Cutis Dyschromica (ACD), which is a rare condition characterized by generalized hyperpigmentation with small hypopigmented macules on the skin (64). Individuals affected by ACD also present reduced levels of GPNMB in the characteristic skin lesions of this condition (64).

**Table 2:** Outcome of models that modulate GPNMB expression *in vitro* or *in vivo*.

	Expression/Treatment	Cell Type/Tissue	Consequence	Reference
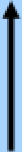	rGPNMB administration *in vitro*	NSC-34 cell line	Protection against SOD1 mutation	(33, 34)
			Activation of PI3K/Akt and MEK/ERK pathways	(34)
		Primary astrocytes	Decreased IL-6 and iNOS levels after LPS treatment	(54)
	rGPNMB administration *in vivo*	Intraventricular injection	Lessened cerebral infarct damage	(37)
	Transgenic mice	Brain	Decreased infarct volume after cerebral IRI	(37)
		Liver	Decreased liver fat accumulation and fibrosis in an obesity model when compared to D2 mice	(63)
	Transfection of GPNMB mRNA	RAW264.7 cell line	Decreased IL-6 and IL-12 levels after IFN-γ/LPS treatment	(52)
		Primary hPDLCs	Decreased TNF-α and IL-12 levels after LPS treatment	(65)
			Decreased apoptosis after LPS treatment	
			Increased IL-10 levels after LPS treatment	
		Human glioma cell line	Increased MMP-3 and MMP-9 levels	(66)
			Increase in metastasis and cancer invasion	
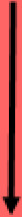	GPNMB siRNA transfection	BV-2 cell line	Decreased iNOS, TNF-α and IL-1β levels after LPS treatment	(51)
		RAW264.7 cell line	Increased expression of *IL-1β*, *IL-6*, *TNF-α* and *MCP-1* mRNA	(62)
			Induces phosporilation of p65 and ERK1/2	
		Primary BM-DCs	Enhanced immunostimulatory capacity	(30)
		DU145 and PC3 cell lines	Upregulation of *MMP-2* and *MMP-9* mRNA	(67)
			Decrease in migration and proliferation rate	
	D2 mice	Colon	More severe colitis	(62)
	Cells obtained from D2 mice	Primary TEPMs	Enhanced upregulation of *IL-1β*, *IL-6*, *TNF-α* and *MCP-1* mRNA after LPS treatment	(62)
			Decreased IL-10 levels after LPS treatment	

The up arrow group (↑), in blue, gathers studies that show the effects of GPNMB by the upregulation of the GPNMB gene by transfection or transgenic mice models or rGPNMB administration. The down arrow group (↓), in red, are studies that evidence the consequences of the lack of GPNMB by using models that knockdown GPNMB expression by siRNA or the use of D2 mice, which has a premature stop codon mutation in the GPNMB gene. hPDLCs, human periodontal ligament fibroblasts; BM-DCs, bone marrow-derived dendritic cells; TEMPs, thioglycollate-elicited peritoneal macrophages.

Diseases that are characterized by macrophage dysfunction also seem to increase GPNMB expression (25). Niemann-Pick type C disease is a condition that arises from lysosomal dysfunction in macrophages and presents increased levels of GPNMB in the liver, brain, and spleen in a mouse model of this disease and in the plasma of patients (59). In another disease characterized by lysosomal dysfunction in macrophages, Gaucher disease, GPNMB is increased in the spleen, plasma (60), and CSF (61) of patients. In a genetically modified mice model of type 1 Gaucher Disease, GPNMB levels were also increased, and after treatment, GPNMB levels returned to normal (60).

Even though pro-inflammatory stimuli lead to increased levels of GPNMB in macrophages and other cell types, not all the cells respond in the same way. In mononuclear phagocytes found in the liver, LPS administration *in vitro* led to diminished levels of GPNMB (57). On the other hand, LPS treatment in macrophages obtained from D2 mice presented increased expression of pro-inflammatory cytokines and diminished levels of IL-10 when compared to macrophages obtained from mice expressing normal levels of GPNMB (62). Also, RAW264.7 cells (macrophage-like cell lineage) transfected with GPNMB siRNA presented increased levels of pro-inflammatory cytokines and p65 phosphorylation when compared with normal RAW264.7 cells (62), indicating that GPNMB might attenuate pro-inflammatory responses. Contributing to the hypothesis that GPNMB could have an anti-inflammatory role, RAW264.7 cells overexpressing GPNMB showed diminished levels of IL-6 and IL-12 after IFN-γ/LPS treatment when compared to the scramble group (52).

Another proof that GPNMB may contribute to the resolution of inflammation is that GPNMB is highly expressed in M2-polarized macrophages rather than M1 macrophages (38, 56), and GPNMB promotes M2 polarization on macrophages (56), which indicates that GPNMB may promote inflammatory resolution. In a periodontal disease cell model, primary human periodontal ligament cells (hPDLCs) overexpressing GPNMB by lentiviral transfection showed decreased pro-inflammatory cytokines TNF-α and IL-12 and increased anti-inflammatory IL-10 levels after LPS treatment (65). In addition, GPNMB protected hPDLCs from apoptosis driven by LPS administration. Increased levels of GPNMB also appear to attenuate other aspects of the disease, not only the ones associated with inflammation. In nonalcoholic steatosis, transgenic mice containing an aP2 promoter-driven GPNMB overexpression showed decreased fat accumulation and fibrosis in the liver in an obesity model when compared to obese D2 mice (63).

Besides interacting with macrophages and monocytes, GPNMB can have a role in the modulation of T cell activation. Activated T cells present receptors to GPNMB, and GPNMB has the ability to inhibit T cells’ response to primary and secondary activation (30). The ability to bind to T cells seems to be dependent on the PKD domain present in the extracellular domain of GPNMB. Endogenous GPNMB prevents T cells from entering the cell cycle, resulting in the inhibition of its activation and proliferation, while GPNMB knockdown T cells display enhanced capacity for immunostimulation (30). Further studies showed that inhibition of T cell activation by GPNMB is dependent on the syndecan-4 pathway (78) and that GPNMB expression regulates lymphocyte allostimullatory capacity, which means the capability of producing antibodies (45). Several studies agree that GPNMB inhibits T cells, which indicates that GPNMB can weaken the immunological response, which is related to both onset and resolution of inflammation.

## GPNMB in Cancer Inflammation

Inflammation is known to predispose the development of cancer and tumor progression (79). Tumor cells can produce cytokines and attract immune cells to the tumor microenvironment, promoting a chronic inflammation at the site of the tumor. The cell types present in the tumor microenvironment may define cancer prognosis and chosen therapeutic approach (80), and the presence of T cells in the tumor microenvironment is required to promote anti-tumoral responses. The ability to inhibit T cell activation may be an important factor that explains the relationship between GPNMB and cancer. Syndecan-4 is an important molecule for the activation of T cells, and GPNMB can bind to syndecan-4, inhibiting T cell activation. A study showed that GPNMB can attenuate the activation of T cells that are activated *via* syndecan-4, allowing the evasion of melanoma cells for immunological recognition and destruction (40), whereas the blockade of GPNMB by antibodies restores the integrity of T cells, attenuates tumor growth, and increases IFNγ levels in the tumor microenvironment (81). Syndecan-4 is expressed in some activated T cells and presents specific heparan sulfates that bind to GPNMB. This interaction leads to a reduction of pro-inflammatory cytokine secretion and blocks T cells from entering the S phase of the cell cycle (45).

One of the currently available cancer therapies is based on immunotherapy drugs called immune checkpoint inhibitors (ICI). Immune checkpoints are a physiological mechanism that prevents exacerbated immune response and cell death, but this mechanism needs to be blocked in cancer to destroy tumorous cells. ICI acts by blocking checkpoints proteins, amplifying the anti-tumoral response, and allowing T cells to kill cancer cells (82). It seems that higher levels of circulating GPNMB in the blood of patients are associated with resistance to ICI therapy (83). Thus, GPNMB seems to modulate the immune response to cancer through several mechanisms.

Another way that GPNMB might contribute to cancer inflammation is by interacting with molecules involved in tumoral progression. GPNMB seems to activate MMP-3 (51), and MMPs activation is involved with both processes that promote cancer cell migration and invasion and inflammation. Overexpression of GPNMB by retrovirus transfection in glioma cells is accompanied by MMP-3 and MMP-9 increase (66), both increasing metastasis and cancer invasion (66). This phenomenon is also documented in other types of cancer, as GPNMB promotes the invasion and proliferation of prostate cancer cell lines through the activation of MMP-2 and MMP-9, which can be reversed by GPNMB knockdown by siRNA transfection (67). It is important to note that, besides cancer, MMPs are also associated with inflammation signaling (84).

## Conclusions

This review shows that GPNMB has a majorly anti-inflammatory role and is important to disease resolution, but there is data that contradicts this tendency. The association of GPNMB with disease resolution is corroborated by the studies that associate GPNMB mutations with a predisposition to Parkinson’s Disease and Autosomal-Recessive Amyloidosis Cutis Dyschromica. However, it is important to notice that GPNMB is associated with cancer progression and metastasis. Regarding inflammation, most of the studies also suggest an anti-inflammatory role of GPNMB, most of them relying on macrophage/microglia activation. As there are also studies suggesting that GPNMB could have a pro-inflammatory role in some cases, more studies should be conducted to clarify this issue. Since GPNMB can interact with different receptors and molecules, the question of whether GPNMB will have a pro- or anti-inflammatory role could depend on which ligand it is interacting with and also the cell type involved.

## Author Contributions

Elaborated the figures and wrote the manuscript: MS and GAS. Reviewed topics and concepts: CS. Conceived, reviewed and discussed concepts in the manuscript: PFK. All authors contributed to the article and approved the submitted version.

## Funding

MS is supported by a Master fellow grant #2018/07896-3, GAS by a student fellow grant #2019/05970-4, and PFK by a postdoctoral fellow grant #2018/14289-6 from São Paulo Research Foundation (FAPESP). CS is a fellow researcher of the National Council for Scientific and Technological Development (CNPq). This publication was made possible by grants from FAPESP to CS (2016/07427-8); CNPq 405089/2018-0; and CAPES – STINT program 88887.125409/2016-00 (Joint Brazilian-Swedish Research Collaboration) and USP Neuroscience Research Support Centres (NAPNA) to CS.

## Conflict of Interest

The authors declare that the research was conducted in the absence of any commercial or financial relationships that could be construed as a potential conflict of interest.
